# Recovering Voiding and Sex Function in a Patient with Chronic Complete Spinal Cord Injury by Olfactory Ensheathing Cell Transplantation

**DOI:** 10.1155/2022/9496652

**Published:** 2022-07-06

**Authors:** Di Chen, Haitao Xi, Ke Tan, Hongyun Huang

**Affiliations:** ^1^Beijing Hongtianji Neuroscience Academy, Beijing 100143, China; ^2^Department of Neurorehabilitation, Capital Medical University Affiliated Beijing Rehabilitation Hospital, Beijing 100144, China; ^3^Department of Neurosurgery, Capital Medical University Affiliated Beijing Chaoyang Hospital, Beijing 100020, China

## Abstract

Spinal cord injury (SCI) is life-altering damage for patients, their family, and society. Transplantation of olfactory ensheathing cells has demonstrated neurorestoration effects for many neurological conditions, including SCI. But voiding and sex dysfunction in patients with chronic complete SCI is still a major issue even though neurorestorative therapies can restore their partial neurological functions. Here we report a case with traumatic complete SCI at the level of C6-C7 one year ago, who received OEC transplantation with intensive neurorehabilitation. The patient started to show clinical improvements within a few days after cell treatment. Six-year follow-up demonstrated his American Spinal Injury Association (ASIA)-Impairment Scale change from ASIA A to become ASIA C. The scores of International Association of Neurorestoratology Spinal Cord Injury Functional Rating Scale changed from 14 (prior to cell therapy) to 31 + 3 (six years after cell therapy). His main improvements in activity of daily life included eating, dressing and writing by himself, standing and walking, and urine control or voiding. His sex function recovered to be normal. He married and had a son through natural sex life. His improving functions and activities of daily life stayed stable in subsequent phone call follow-up. This was one individual case report. In the future, the deep mechanisms of why he got positive results, but other patients with similar condition did not get so much benefits from OEC transplantation should be explored.

## 1. Introduction

Spinal cord injury (SCI) is life-altering damage for patients, their family, and society. Olfactory ensheathing cells (OECs) were transplanted for patients with SCI to restore injured cord functions and structures 20 years ago [1]. Cellular therapies have been found to have many benefits in clinical studies. Most patients with SCI got various benefits from OEC therapy [2–16], but voiding and sex dysfunction in patients with chronic complete SCI is still a major issue. Here we present a case report in which an adult young male with one year completes SCI received OEC transplantation. Besides improvements of motor function, sensation, and activity of daily life, he recovered his voiding and sex function during long-term follow-up.

## 2. Case Presentation

### 2.1. A Case Report

**Figure 1:**
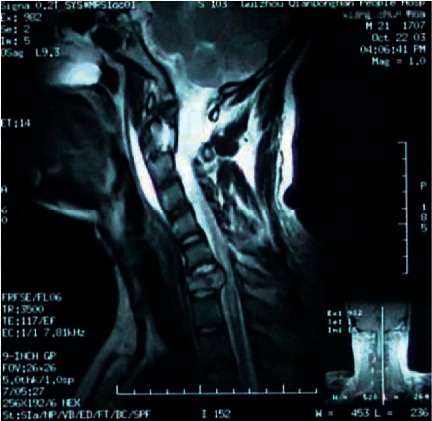
The spinal cord was compressed by fracture vertebral body.

A 21-year-old man was injured in a car accident one year ago, causing trauma to the spinal cord. His lower limbs and hands were paralyzed after the accident. Magnetic resonance imaging (MRI) showed focal cord compression and injury by fractured bone and protruded disc in C6 (see Figure 1). He suffered spinal cord decompression and spinal stabilization. After extensive rehabilitation, his hand functions partially recovered. However, his functional recovery reached plateau and stopped recovering losing functions for half year before requesting for our treatment.

**Figure 2:**
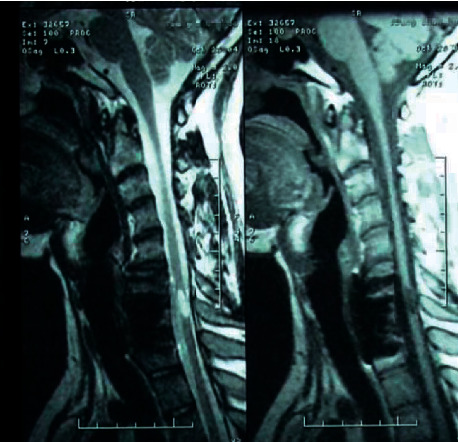
The cavitation volume is about 1 × 0.5 × 0.5 cm^3^ in cervical vertebral 6.

Prior to cell transplantation, his sensory level intact was at C6 for pinprick and light touch bilaterally, abnormal sensation at C7, and no sensation in other dermatomes below T1. Both sides' muscle testing showed grade 4 at C7 function and no voluntary muscle movement below the T1 level. The rectal exam indicated no sensory or motor function preserved in sacral segments S4/5. Therefore, the deficit was classified as American Spinal Injury Association (ASIA)-Impairment Scale was at level C7 ASIA A motor and sensory complete tetraplegia. The scores of International Association of Neurorestoratology Spinal Cord Injury Functional Rating Scale (IANR-SCIFRS) were 14 out of 48. His activities of daily living were difficult, such as eating, dressing, standing, walking, and poor trunk control. He had urinary incontinence with poor urine control. MRI showed focal myelomalacia from C6 to C7 with altered signals at these two levels (see Figure 2).

### 2.2. Cell Preparation and Transplantation

Olfactory ensheathing cells (OECs) were isolated from aborted human fetal olfactory bulbs (received approval and signed donation consent form). The OECs were cultured in Dulbecco's modified Eagle's medium/Ham's F12 (DMEM/F12; Hyclone, Logan, UT, USA) with 10% fetal bovine serum (FBS; Hyclone) and the neurotrophic factors and then propagated for 2-3 weeks. The cells were characterized by immunostaining with antibodies against p75 (Sigma, St. Louis, MO, USA).

A signed informed consent was obtained from the patient and his family member. The protocol of cell therapy was approved by the ethics committee. Under general anaesthesia, the patient underwent cervical posterior approach surgery to expose injured cord in C6 and 7. OECs (1 × 10^6^ in 0.06 ml DMEM liquid) were transplanted into the spinal cord parenchyma in adjacent upper and lower injured site (the injecting OEC amount and delivery vehicle was referred to our pre-clinical research in rats [17]). Cell injection procedure is under microscopy, injection points are in the middle of the upper and lower injured cord borders with normal tissue, injection rate is slowly (each injection takes about 10 seconds), the depth of injection is about 3-4 mm within cord parenchyma. After cell injection is completed, the needle is kept for 2-3 seconds. The gauge size of this needle is 4.5^#^ thin needle (Chinese product specifications). The patient recovered well after the surgery and cell transplantation without using immunosuppression. Then, he continued intensive neurorehabilitation as before.

## 3. Results

He started to show some clinical recoveries of lost neurological functions in a few days after cell transplantation, including sensory and motor improvement in hands, trunk, and lower limbs.

Assessments were conducted at three weeks after cell transplantation. His sensation, motor functions, and activity of daily life improved. His ASIA AIS was A. ASIA motor scores were 42; ASIA pin prick scores were 34; ASIA light touch scores were 40. IANR-SCIFRS were 17.

Assessments conducted in six-year follow-up showed improving sensation, motor functions, and activity of daily life. His ASIA AIS changed from Grade A to Grade C; ASIA motor scores were 54; ASIA pin prick scores were 66; ASIA light touch scores were 82. IANR-SCIFRS were 31 + 3 (sex function)/48. The main recoveries took in upper limb, trunk, and lower limb movement. He could finish eating, dressing, and writing by himself. With brace and walker support, he could stand and walk. His rectal function had improved. He was able to hold urine and void almost normally without any assistance. His sex function recovered to be normal (married and had a son through natural sex). His improving functions and activities of daily life remained stable in subsequent phone call follow-up.

## 4. Discussion

### 4.1. Managing Voiding and Sex Dysfunction in Clinic

Condom drainage for voiding remains the most frequent bladder method for male patients with spinal cord injury [18]. Clean intermittent catheterization is also a preferred method of bladder management for many patients with SCI, but long-term adherence is low [19]. Patients could undergo neurorestorative treatments, such as nerve bridging, neurostimulation/neuromodulation, cell therapy, and so on, for their voiding [20]. There are some methods for ejaculation in SCI patients with sex dysfunction. Electroejaculation is suitable for the injured level at caudal to T10. Ejaculation in penile vibratory stimulation is appropriate for men with SCI at T10 or rostral [21]. In vitro fertilization/intracytoplasmic sperm injection is for male patients with SCI, who want to have children [22]. So far, there are no reported methods for man with chronic complete SCI to recover their natural sex function and have children except this report.

### 4.2. OEC Transplantation for Patients with Spinal Cord Injury

Our team first transplanted OEC into the spinal cord parenchyma in adjacent injured site for SCI patients in 2002. Twenty-three patients with chronic SCI (most complete) improved their neurological functions and quality of life and still kept the improving trend after OEC transplantation during follow-up [1]. Later, the study of long-term follow-up by our team proved the safety and effects of OEC transplantation for patients with SCI [3]. Important finding was that chronic complete SCI patients with intensive neurorehabilitation received more benefits than patients with poor neurorehabilitation [3]. Simultaneously, more clinical studies of OECs or their combination with other cells had been reported, in which patients with acute, subacute and chronic, complete, and incomplete SCI at different injured levels demonstrated neurological functional improvements including sensation, motor, urine and rectal control, and activities of daily life [2, 4–16].

### 4.3. Neurorestorative Mechanisms of OECs

OECs are special glial cells sharing the characteristic of Schwann cell and astrocyte properties. Their neurorestorative mechanisms include neuroprotection, axonal regeneration, remyelination, neural network or circuitry reconstruction, neuroplasticity, neuromodulation, anti-inflammatory response or immunomodulation, promoting neurogenesis, stimulating angiogenesis, and so on [23]. There is no barrier for OECs between the central nervous system and peripheral nervous system. They can easily migrate into the injured spinal cord after being transplanted and restore injured nerves which are responsible for voiding and sex function. This patient's voiding and sex function may be restored through those mechanisms.

### 4.4. Limitation and Unsolved Questions

Among our treated patients with chronic complete SCI, one third of them recovered more or less voiding and rectal functions. Also male patients recovered partially sex function, but few patients could recover to be normal state like this reported patient. The reasons puzzle us, which need further exploration.

## 5. Conclusion

OEC transplantation with intensive neurorehabilitation showed favorable effects in improving neurological function in this patient with chronic complete SCI, especially in recovering his voiding and sex function. His improving functions and activities of daily life remained stable in subsequent phone call follow-up. Since this was a single case report, not all patients could attain positive results like this patient. In the future, the deep mechanisms, and the reason why some patients received more benefits and some received fewer benefits from OEC transplantation, should be explored.
